# Supplementary material for: Reproductive coercion and abuse in intimate relationships: Women’s perceptions of perpetrator motivations
Source: PLoS One. 2024 Apr 16;19(4):e0299069. doi: 10.1371/journal.pone.0299069 (PMC11020648; doi:10.1371/journal.pone.0299069)
Supplement: Attachment — Submitted filename: Response to reviewers.docx [file pone.0299069.s001.docx]

Dear Editors,

Thank-you for the opportunity to revise our manuscript. We are greatly appreciative of the kind and thoughtful feedback provided by the two reviewers. We have done our best to address all of their comments, as well as the additional editorial points. These are detailed below.

**Editors’ comments**

1. Please ensure that your manuscript meets PLOS ONE's style requirements, including those for file naming.

We have amended the formatting to align more closely with the PLOS ONE style requirements. Please let us know if further changes are required.

2. Note from Emily Chenette, Editor in Chief of PLOS ONE, and Iain Hrynaszkiewicz, Director of Open Research Solutions at PLOS: Did you know that depositing data in a repository is associated with up to a 25% citation advantage (<https://doi.org/10.1371/journal.pone.0230416>)? If you’ve not already done so, consider depositing your raw data in a repository to ensure your work is read, appreciated and cited by the largest possible audience. You’ll also earn an Accessible Data icon on your published paper if you deposit your data in any participating repository (<https://plos.org/open-science/open-data/#accessible-data>).

This is not appropriate for our very sensitive qualitative dataset (see point 4 below).

3. Please remove any funding-related text from the manuscript and let us know how you would like to update your Funding Statement…Please include your amended statements within your cover letter; we will change the online submission form on your behalf.

We have removed the funding-related text from our Acknowledgements in the manuscript. No updates are required to the Funding Statement. We have added the amended statements to our cover letter as requested.

4. In the online submission form you indicate that your data is not available for proprietary reasons and have provided a contact point for accessing this data. Please note that your current contact point is a co-author on this manuscript. According to our Data Policy, the contact point must not be an author on the manuscript and must be an institutional contact, ideally not an individual. Please revise your data statement to a non-author institutional point of contact, such as a data access or ethics committee, and send this to us via return email. Please also include contact information for the third party organization, and please include the full citation of where the data can be found.

We have amended the contact point to the administrator of our University’s Human Ethics Committee, with the caveat that any person seeking to utilise the data would need to meet the criteria for access. We note, however, that we would be extremely reluctant to release the interview transcripts to others. There is a certain amount of trust between researcher and participant in undertaking this sort of research, and the participants did not consent to the use of their data for any purpose other than our own study. Moreover, due to the subjective nature of qualitative analysis, we feel that the benefits of sharing raw data are limited. Different people will always have different interpretations of the data, no matter how rigorous the analysis. If there is any way that we can waive the data access requirement, this would be the ideal situation for us, but we understand that this may go against the editorial policies and may not be possible.

5. Please review your reference list to ensure that it is complete and correct. If you have cited papers that have been retracted, please include the rationale for doing so in the manuscript text, or remove these references and replace them with relevant current references. Any changes to the reference list should be mentioned in the rebuttal letter that accompanies your revised manuscript. If you need to cite a retracted article, indicate the article’s retracted status in the References list and also include a citation and full reference for the retraction notice.

We have reviewed the references and as far as we can tell it is complete and correct. We have not cited any retracted papers. We have edited some references only for formatting.

**Response to reviewers' comments:**

**REVIEWER #1**

| The introduction is intriguing but revolves solely around the subject of study, overlooking the concept of motivation. However, numerous criminological theories address the issue of criminal motivation. It is important to note that several studies indicate that perpetrators of intimate partner violence are generalists rather than a distinct criminal category. A recent synthesis by Koegl & Farrington (2022) highlights 17 motivations. In his situational action theory, Wikström (2014) suggests that a crime is the chosen action of an individual in response to a specific situational motivation. Motivation is conceptualized as the result of the interaction between the individual (preferences, sensitivities, and commitments) and the situation (opportunities, constraints). Deci & Ryan (2002) identify different motivational orientations associated with varying levels of self-determination, offering a useful conceptual framework for understanding the studied phenomenon. Intrinsic motivation refers to engagement in an activity for the inherent pleasure and satisfaction derived from it, resulting in high self-determination. Extrinsic motivation occurs when engagement in a behavior depends on instrumental reasons, indicating lower self-determination. Finally, the construct of amotivation refers to the absence of motivation and represents the lowest level of self-determination. In my opinion, it would be crucial to review the conceptualization of the motivation concept | Thank-you for this feedback. The suggestion made here represents a very criminological view of the topic, and whilst we completely respect the value of this approach as one possible option, it is not the lens through which we have chosen to examine RCA. It is important to note that RCA itself is not considered to be a “crime”, and many of the behaviours reported by participants could be seen (at least from the outside) to fall within the grey area of “normal” relationship behaviours. Therefore, it is our view that much of the literature on what motivates criminals is not relevant. Most of these perpetrators would not have thought they were perpetrating any sort of “crime”, and therefore considerations about cost vs benefit etc do not apply.  Having said this, we agree that it would strengthen the manuscript to review the literature on perpetrator motivations for IPV more broadly, and the psychological concept of motivation as suggested. We have added a sub-section to the introduction to this effect. |
| --- | --- |
| The theoretical framework (ecological feminist framework) should be presented in greater detail (proposals, principles). | We have removed the reference to the ecological model, since ultimately, our analysis did not focus on factors at the level of individual, relationship, community, and society. Rather, we took a broader feminist approach that recognises the gendered nature of RCA, sexual and IPV, whilst also considering situational and individual factors. We have clarified this in the manuscript. |
| I'm curious to know if there's any information available regarding the victimization trajectories of these women within the context of intimate relationships, especially prior to the incidents of (RCA). This includes details on the forms of violence, duration, and any relevant information. Specifically, I'm interested in understanding whether the dynamics of violence have undergone any changes during pregnancy. | Whilst we agree that this would be interesting, it is outside the scope of this manuscript to provide individual victimisation trajectories for each of the 30 participants. We have added some sentences to the beginning of the Findings section explaining that women reported different trajectories of violence. Some reported pregnancy and RCA in the context of pre-existing physical and sexual abuse, whilst for other participants, the pregnancy was the trigger for violence to start. |
| Examining the motivation of a perpetrator of domestic violence solely based on his partner's perception may introduce several potential challenges and limitations to the research. Here are some considerations: 1. Subjectivity and Bias: Relying solely on the victim's perspective can introduce subjectivity and potential bias into the analysis. The victim may have a subjective interpretation of the perpetrator's motivations, influenced by emotional distress, fear, or personal experiences. 2. Incomplete Picture: The victim may not have access to the complete range of the perpetrator's motivations. Certain factors or aspects of the perpetrator's psychology, background, or external stressors might not be fully known or understood by the victim. 3. Lack of Cross-Verification: Relying solely on one perspective limits the ability to cross-verify information. Including multiple perspectives, such as the perpetrator's own account or insights from other sources, can enhance the reliability and validity of the findings. 4. Complexity of Motivation: Understanding human motivation is inherently complex. Motivations for abusive behavior can be multifaceted and influenced by a range of personal, psychological, social, and situational factors. A comprehensive analysis requires a nuanced and multifactorial approach.  To address these challenges, a more comprehensive research approach might involve triangulating information from multiple sources, such as interviews with both partners, observations, and perhaps external records or reports. This holistic approach can provide a more nuanced and accurate understanding of the motivations behind domestic violence. In my opinion, the part in the limits in the discussion (2nd limit) that deals with this should be improved. | We agree that the partner’s perspectives cannot tell us exactly what the perpetrator was thinking, providing an incomplete or biased version of events. We did already acknowledge this in the Limitations section, however, we have now strengthened this so that it addresses these issues in more detail.  Although triangulating the women’s accounts by interviewing the perpetrator would indeed provide a more “accurate” picture of the perpetrator’s motivation, this would obviously not be safe for the victim/survivor. It is also important to note that many of the women did not have any contact with the justice system, and so no other data sources exist to “verify” their accounts. We feel that, as a qualitative study, it is not necessary to present the data as objective “truth” – what we have reported are the perceptions of the women as told in their own words. Our role is to reflect on and interpret their stories, whilst being careful not to make claims that are overblown. We hope that we have achieved this! |
| **REVIEWER #2** |  |
| I find the title overly long. I suggest removing “and overlaps with coercive control.” This phrase is confusing for the reader who is not an expert in IPV literature. | We have removed “and overlaps with coercive control” and replaced it with “Women’s perceptions of perpetrator motivations” |
| I think the themes should be included in the abstract. | We have added them. |
| Pg. 3, line 54: RCA is “conceptually contested.” Please explain how and why. | We have removed this part of the sentence, since this point was already made in the preceding paragraph. |
| Pg. 3, line 57: RCA rates of 8% and 30%. Percent of what? All adult women (where)? Mothers? Women who experience IPV?... | We have clarified these percentages. |
| Pg. 3, line 60: How does national income level affect risk factors? | We are not making claims about the impact of national income level on risk factors. We are simply reporting that studies in low-income settings (e.g. Bangladesh, Sub-Saharan Africa) have identified different risk factors compared to high-income settings. It may be that other cultural or community level factors are more relevant than (or inter-related with) the economic ones. |
| Pg. 5, line 102: There seems to be a contradiction here. The authors state that, “there is little data regarding motivations for RCA, particularly in high income countries.” They then point out that the “evidence base overall is strongly weighted in favor of U.S. and similar settings.” Isn’t the U.S. a high income country? Perhaps the order of the two sentences should be reversed. Also, what is a setting “similar to the U.S.”? | The sentence reads: “Overall, however, there is very little data to help understand *why* men perpetrate RCA against their intimate partners, particularly in high-income countries (**in contrast to the RCA evidence base overall**, which is strongly weighted in favour of the US).”  We are pointing out that although most of the RCA evidence overall comes from the US, on this particular aspect (understanding motivations), there is little evidence from high-income countries. We have removed the “similar to the US” part of the sentence, since most of the literature is US-based. |
| Pg. 7, line 147: I understand that the findings presented here are part of a larger study that also dealt with the perceived impact of RCA on mothers’ relationships with their children. Were all of the participants mothers? What about women whose partners pregnancy prevention practices left them without children? | All participants were mothers. Those who had been forced to terminate a wanted pregnancy in the past had gone on to have other children (in most cases with a new partner). Our forthcoming manuscript explores how historical RCA could continue to impact women’s relationships with their other children for many years. |
| Please state that none of the women was currently living with the partner who had been abusive. | One participant was still living with the partner who had perpetrated the RCA. We have now stated this at the beginning of the Findings section. |
| Pg. 7, line 162: How many of the interviews were conducted in on-line video conferencing and how many in telephone calls? Why not do video conferencing for all interviews? The authors state that there were 30 participants and 33 interviews. Were three participants interviewed twice? If so, why? Where three interviews/participants excluded from the study? If so, why? | Only one interview was conducted using online video conferencing. We have had numerous experiences with imposter/fraudulent participants using online methods (ie participants outside of Australia who do not meet eligibility criteria) and so prefer to use telephone unless the participant indicates a preference for video conferencing. All participants were interviewed only once. The [33] is an in-text citation, not an indication of the number of interviews. To avoid confusion, we have removed the citation (it appears a few lines below in any case) and added (n=29) and (n=1) respectively for each interview method. |
| Pg. 9, line 202: “In the event of participant distress, a standard protocol was developed to guide responses.” Please provide details of this protocol and how it was developed. | We have provided further details about the distress protocol. |
| Pg. 10, line 213 : What does “primarily educated” mean? | It means the majority of participants were educated, born in Australia etc… We have rephrased to avoid confusion. |
| I’m not sure that the title of the third theme captures its essence. How about, “A real man must create and illusion of the perfect father.”  Also, at some point (either in the introduction, at the beginning of the findings section, or at the beginning of this theme) you should note that RCA comprises not only fathering a child, but also of the way one enacts fatherhood. This is apparent in the third and fourth themes. | Thanks for this suggestion. We have given it careful thought, but ultimately we prefer to keep the third theme title as it is. Although some participants felt that their partners were motivated by insecurity about their masculinity, this was not the case for all participants. We feel that the broader theme title allows space for the other explanations provided by participants.  RCA does not comprise the way one enacts fatherhood. Although the third and fourth themes contain detail about how the men enacted fatherhood after perpetrating RCA, this is not part of the RCA itself. Rather, it explains how the RCA is part of a broader pattern of coercive control in the relationship and towards his children. |
| I suggest adding a summarizing sentence at the end of the findings section. | We have added a sub-section “Summary of findings”. We have moved Fig.1 out of the Discussion and into this section. |
| Pg. 23, line 556: Not all of the readers are familiar with Evan Stark and his writings. If you wish to highlight him, situate him within the literature on IPV and cite his definition of coercive control (consider placing it in the introduction of the article) or make it clear that you are using his definition as a working definition for your research. | Thanks – this is an important point. We do already have a definition of coercive control as described by Evan Stark in the Introduction (see page 5, lines 87-89), however, we have made this clearer and explicitly stated that we are using his definition in our research. |
| Pg. 25, line 584: “The situational couple violence in the study was not mutual.” Add “according to the participants.” Were the women aske about their use of coercion in the relationship? | We have added “according to the participants in this study”. We did not ask the women specifically about their use of coercion in the relationship, however, they were asked to provide detailed background about the relationship and the context for the RCA, in which such details are likely to have emerged. |
| Pg. 621, line 621: “IPV, SV” Abbreviations should not be used the first time a term is mention. | We have removed the abbreviations |
| Pg. 29, line 684: Change “interrogating” (which is a criminal term) to “exploring” (which is a qualitative research term). | This has been changed. |
|  |  |
